# Continuous Infusion of Ketamine in Mechanically Ventilated Patients with SARS-CoV-2

**DOI:** 10.1155/2024/7765932

**Published:** 2024-05-10

**Authors:** Brian Phan, Afua Agyemang, Walter Klein, Suman B. Thapamagar

**Affiliations:** ^1^Department of Pharmacy, Riverside University Health System, Moreno Valley, California, USA; ^2^Department of Internal Medicine, University of California, Riverside School of Medicine, Riverside, California, USA; ^3^Department of Pharmacy, Allegheny General Hospital, Pittsburg, Pennsylvania, USA; ^4^Department of Medicine, Riverside University Health System, Moreno Valley, California, USA; ^5^Department of Medicine, Loma Linda University School of Medicine, Loma Linda, California, USA

## Abstract

**Background:**

Widespread drug shortages led to higher utilization of ketamine in our intensive care unit, especially among patients with SARS-CoV-2.

**Objectives:**

To evaluate the impact of continuous infusion of ketamine on vasopressor requirements in patients with SARS-CoV-2.

**Method:**

This was a single-center, retrospective, cohort study comparing mechanically ventilated (MV), adult patients with SARS-CoV-2 receiving either propofol or ketamine for at least 72 hours.

**Results:**

84 patients (mean age of 61-year-old, 68% male) were analyzed. 31 patients received ketamine, and 53 patients received propofol. Mean vasopressor doses were not significantly different between ketamine and propofol groups at prespecified timepoints. However, mean arterial pressures (MAP) were higher in the ketamine group at 24 h, 48 h, and 96 h postsedative initiation. The median opioid infusion requirements were 3 vs. 12.5 mg/hr (*p* < 0.0001) for ketamine and propofol groups, respectively. Comparing to propofol, C-reactive protein (CRP) values were significantly lower in the ketamine group at 24 h (7.53 vs. 15.9 mg/dL, *p*=0.03), 48 h (5.23 vs. 14.1 mg/dL, *p*=0.0083), and 72 h (6.4 vs. 12.1 mg/dL, *p*=0.0085).

**Conclusion:**

In patients with SARS-CoV-2 on MV, there was no difference in the vasopressor requirement in patients receiving ketamine compared to propofol. Nevertheless, the use of ketamine was associated with higher MAP, reductions in CRP in select timepoints, and overall lower opioid requirements.

## 1. Introduction

Mechanically ventilated (MV) patients often require sedation to help manage their agitation, ventilator dyssynchrony, and potential harm to self and/or others. Propofol and dexmedetomidine are preferred by most clinicians and typically used as first-line agents [1]. These agents, however, do come with their own sets of adverse events. Propofol is known to cause hypotension, and at higher doses, with prolonged infusion, propofol-related infusion syndrome [2]. The use of dexmedetomidine can lead to bradycardia and does not, on average, achieve a deep level of sedation [3]. Ketamine, an N-methyl-D-aspartate (NMDA) receptor antagonist, has shown some potential benefits as a sedative in critically ill patients due to its favorable hemodynamic profile as well as opioid and benzodiazepine sparing effects [4]. It inhibits the reuptake of catecholamines and acts on sigma-opioid receptors. As a result, ketamine can produce psychotomimetic and analgesic effects, making it an attractive option in multiple clinical scenarios [5, 6]. Emergence reaction and hypersalivation are often cited as clinically important side effects from the use of ketamine as a sedative [7]. Nevertheless, studies have shown ketamine to be a safe and effective sedative comparable to other agents in critically ill patients [8]. In select studies, the use of ketamine has been shown to reduce time to achieve targeted sedation goal and overall administration of opioids and other sedatives [7–9]. Regarding its hemodynamic effects, studies have yielded mixed results on ketamine being vasopressor-sparing and/or leading to higher mean arterial pressures (MAP) [8–12].

As our local healthcare system started facing multiple drug shortages (including propofol and dexmedetomidine) due to the Coronavirus Disease 2019 (COVID-19) pandemic, the use of ketamine in the intensive care unit (ICU) as a sedative became necessary. The reemergence of the use of ketamine in patients with severe acute respiratory syndrome coronavirus 2 (SARS-CoV-2) infection also brought about a new clinical question to investigate. In animal models, ketamine has been implicated in potentially reducing interleukin-6 (IL-6), TNF-alpha, and other proinflammatory markers (e.g., C-reactive protein (CRP), ferritin), all of which are found to be elevated in critically ill patients with SARS-CoV-2 [13, 14]. However, the effects of ketamine on these specific markers in patients with severe SARS-CoV-2 are yet to be determined.

Considering the potential benefits of ketamine in critically ill patients with SARS-CoV-2, this study sought to compare the effects of ketamine to propofol as a primary sedative in these MV patients. We hypothesized that the use of ketamine would favorably impact patients' hemodynamics and reduce opioid requirements along with our inflammatory markers of interest (CRP and ferritin).

## 2. Methods

We conducted a retrospective cohort study of adult patients admitted to the medical ICU between April 1^st^, 2020, and February 28^th^, 2021, at Riverside University Health System-Medical Center (RUHS-MC), a safety-net hospital in Moreno Valley, California. These dates were chosen as they coincided with the shortage of traditional agents and, consequently, the most frequent use of ketamine as an alternative sedative. The research protocol was approved by the Institutional Review Boards at the Western University of Health Sciences and RUHS-MC. MV patients with a laboratory-confirmed diagnosis of SARS-CoV-2 infection who received continuous sedation with either ketamine or propofol for at least 72 hours were included. Patients were excluded if they received ketamine for other indications (e.g., procedural sedation), crossed over between ketamine and propofol, or had baseline neuropsychiatric diagnosis to avoid any psychiatric events being miscategorized as reemergence reactions due to ketamine. The study timeframe of eligible patients was from hospital admission until ICU discharge or death, whichever occurred first.

Primary outcomes of this study were vasopressor requirements in norepinephrine equivalent (NE) in micrograms per minute (mcg/min) at 24 hours (24 h), 48 h, and 72 h after sedative initiation. Secondary outcomes included CRP and ferritin levels at 24 h, 48 h, and 72 h after sedative initiation; opioid infusion requirements at 24 h, 48 h, 72 h, and 96 h after sedative initiation; and cumulative dosing of intermittent opioid administration in morphine equivalent (ME) in milligram (mg). The incidence of reemergence reaction to ketamine; MAP at 24 h, 48 h, 72 h, and 96 h after sedative initiation; RASS compliance; ICU length of stay (LOS); and in-hospital mortality were also evaluated as additional secondary outcomes.

### 2.1. Data Variables

All data points were abstracted from our electronic medical record system. Potentially eligible patients were identified by running a report of patients using “ketamine” or “propofol” as keywords during the aforementioned timeframe using Vigilanz, a clinical intelligence software package at RUHS-MC. The list of patients was then manually evaluated to confirm study eligibility. Conventional key demographic data were collected (e.g., age, sex, baseline comorbidities). All baseline data were from the first available set of data during their hospital admission. Obesity was defined as having a body mass index (BMI) above 30 kg/m^2^. We also collected all components of the Acute Physiologic Assessment and Chronic Health Evaluation II (APACHE II) score. Vasopressor use was defined as the administration of one or more of the following agents: norepinephrine, epinephrine, phenylephrine, or vasopressin. Furthermore, all recorded vasopressor doses at select timepoints were converted into non-weight-based norepinephrine equivalent (NE) in microgram/minute (mcg/min). MAP values were obtained from arterial line whenever possible throughout the study. Opioid use data were collected using both the continuous infusion at the select timepoints and total cumulative dose of intermittent administration to capture total utilization. Average opioid continuous infusion was the average of all five infusion rates (i.e., baseline, 24 h, 48 h, 72 h, and 96 h after sedative initiation). All opioid doses were converted into ME. Ketamine-associated reemergence reaction was defined as an episode of nightmare, agitation, and/or hyperactive delirium among patients whose ketamine anesthetic effects were waning [9]. In our study, it was determined by the receipt of any antipsychotics or benzodiazepine and nursing documentation of evidence of hallucination or agitation. RASS goals and compliance were recorded at the baseline, 24, 48, and 72 h after sedative initiation. Compliance was determined by the proportion of patients whose documented RASS values matched the intended RASS goal by the prescribing physician. Study data were collected and managed using Research Electronic Data Capture (REDCap) tools hosted at RUHS-MC.

### 2.2. Statistical Analysis

Categorical variables were analyzed using the chi-squared test. A normality test was performed on all continuous data using the Shapiro–Wilk test. Normally distributed continuous variables were reported as the mean and standard deviation and analyzed using a two-sided Student's *t*-test. Nonparametric continuous data were reported as the median and interquartile range and analyzed using the Wilcoxon rank-sum test. The Wilcoxon signed-rank test was used for paired comparisons in the ketamine group. An observed *p* value of less than 0.05 was considered statistically significant. Data were analyzed using STATA software (version SE 16). With an 80% power, two-sided *α* of 0.05, and a 2 : 1 enrollment ratio, our calculated sample size would be 41 and 20 participants for the propofol and ketamine arms, respectively, to appreciate a 20% reduction in vasopressor requirements, which was deemed clinically significant to us. Multivariate linear regression analyses were performed to determine the effects of ketamine on vasopressor requirements, MAP and CRP values at select timepoints.

## 3. RESULTS

**Figure 1:**
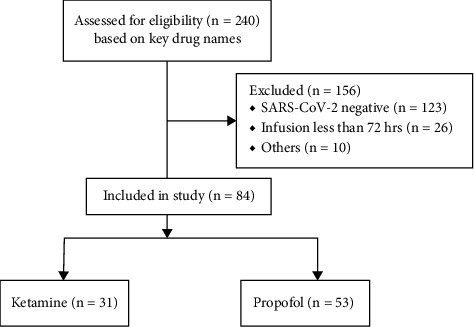
Study flowchart.

A total of 240 patients were identified by Vigilanz; of these, 130 and 110 patients received propofol and ketamine, respectively. The study flow diagram (Figure 1) summarizes the reasons for exclusion from the study (156 patients). Among the ten patients with other reasons listed, seven had a baseline psychiatric diagnosis and three had crossover infusions between ketamine and propofol for less than 72 hours. A total of 84 patients were included in the final analyses. The population characteristics are summarized in the Table 1.

Most patients received corticosteroid as part of their overall management (96.8% and 92.5% in ketamine and propofol groups, respectively); most likely, hydrocortisone was given as stress-dose steroids given the study timeline. Patients' demographic and clinical characteristics did not significantly differ between the groups, except for the higher proportion of patients with concurrent use of dexmedetomidine in the ketamine group (62% vs. 28%, *p*=0.003). Additionally, patients on ketamine were noted to have a higher median APACHE II score (25 vs. 22, *p*=0.017). This may also explain the higher proportion of patients receiving vasopressors at the baseline with a higher mean vasopressor dose in the ketamine group (8.95 mcg/min) compared to those on propofol (3.82 mcg/min, *p*=0.043).

**Figure 2:**
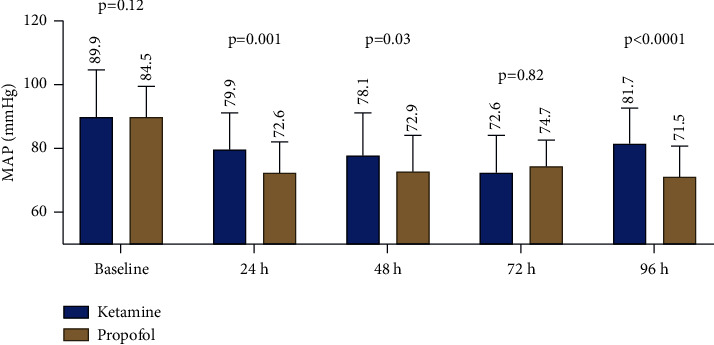
Mean arterial pressures at different timepoints between ketamine and propofol groups.

There were no differences in vasopressor requirements between ketamine and propofol groups at 24 h, 48 h, and 72 h (Table 2). MAP in the ketamine group remained significantly higher than those of the propofol group at all select timepoints, except at 72 h (Figure 2). CRP values were significantly lower among patients on ketamine at 24 h, 48 h, and 72 h. Furthermore, patients who received ketamine received less opioids as continuous infusion compared to propofol across 24 h, 48 h, 72 h, and 96 h. The average opioid infusion rate for ketamine was 3 mg/hr (IQR 0–16) as compared to 12.5 mg/hr (IQR 7–16.5) in the propofol group (*p*=0.015). The incidence of agitation was found in 42% of patients on ketamine compared to 21% of those on propofol (*p*=0.04). There were no significant differences in ferritin levels, RASS compliance, and mortality between the two groups. However, patients on ketamine had a longer ICU LOS compared to the propofol group (18 vs. 9 days, *p*  <  0.0005, respectively). See Table 3 for all secondary outcomes of the study. Multiple multivariate analyses were conducted incorporating critical variables impacting MAP and CRP values. Ketamine infusion was associated with a higher MAP goal at 24 h and lower CRP values at 48 h and 72 h.  Table 4 highlights select multivariate linear regression analyses. Complete sets of these analyses can be found in the supplemental materials (available here).

## 4. Discussion

Among critically ill patients with SARS-CoV-2 on MV, our study found no significant differences in vasopressor requirements between patients on ketamine and propofol at 24 h, 48 h, and 72 h timepoints. This is consistent with select studies showing no impact of ketamine use on vasopressor requirements [7, 11, 15, 16]. Other existing studies, however, do demonstrate the vasopressor-sparing effect of ketamine [10, 12, 17]. We suspect that the variations in dosing and patient heterogeneity along with small sample sizes are to blame for this inconclusiveness. When evaluating MAP at the select timepoints, patients in the ketamine group demonstrated significantly higher mean readings at 24 h, 48, and 96 h. When adjusted for agitation, baseline dexmedetomidine use, vasopressor dosing, and APACHE-II score in a multivariate linear regression model, MAP remained significantly higher in the ketamine group at 24 h. This could be due to the known ability of ketamine in blocking catecholamine reuptake causing tachycardia and hypertension [5, 8]. As to why higher MAP in the ketamine group did not translate to lower vasopressor requirements in our study, we suspect this was primarily due to lack of timely titration of vasopressors. Nonetheless, the positive hemodynamic attribute of ketamine is one of the primary reasons for its reemerging use among critically ill patients. Our study also corroborated the opioid sparing effects of ketamine from the existing literature [8]. At our institution, continuous infusion of fentanyl was often run concurrently with a sedative to target a deeper sedation level and optimize patient-ventilator synchrony. The deeper sedation goal seen in our study was reflective of our overall management strategy at the time for patients with acute respiratory distress syndrome associated with severe SARS-CoV-2 given the high census, lack of adequate self-proning beds, and intermittent shortages of neuromuscular blockers. With a significantly lower dose of opioids being used in ketamine patients (3 mg/hr vs. 12.5 mg/hr in ME), this could help mitigate opioid-induced adverse events and serve as an alternative in the event of a drug shortage.

Ketamine and dexmedetomidine both have been associated with anti-inflammatory effects [18, 19]. We noted lower CRP values in the ketamine group across three different timepoints. When adjusted for the concurrent use of dexmedetomidine among two groups, these findings still hold true at 48 h and 72 h among patients receiving ketamine. To our best knowledge, our study is the first one to report the effect of ketamine on a key inflammatory marker such as CRP. While IL-6 and CRP were found to be significantly reduced with the intraoperative use of ketamine in surgical patients, its mechanism and applicability to other populations remain unclear [19]. CRP and other inflammatory markers may have a prognostic value in patients with SARS-CoV-2, even though evidence to support a specific marker with a specific threshold remains unclear [20]. One study demonstrated the strong association between elevated baseline CRP and the development of venous thromboembolism, acute kidney injury, critical illness, and even mortality [21]. Pata and colleagues attributed to the mortality benefit of ketamine in their patients with SARS-CoV-2 to its antioxidant and anti-inflammatory properties [22]. At our institution, during the timeframe of this study, ferritin and CRP were routinely checked to evaluate for evidence of cytokine release syndrome and hence chosen as our secondary outcomes. It would have been of great interest to evaluate the impact of ketamine on IL-6 given its more direct implication in therapeutics [23]. However, IL-6 was not available for in-house processing at our institution. When evaluating LOS between the two groups, patients in the ketamine group had a longer stay in the ICU. This could be attributed to the higher APACHE-II score at the baseline of these individuals. High mortality rates were similarly found in both groups and could be attributed to high severity of disease at the baseline and lack of therapeutic options with survival benefit at the time the study was launched.

While the use of ketamine did not have a negative impact on the RASS compliance, it did lead to an increase in the incidence of agitation. The theoretical risk of ketamine reemergence phenomenon has always been concerning given ketamine's various receptor- and dose-dependent effects. However, the actual reported incidence of ketamine-induced delirium/psychosis in MV patients has not been found to be statistically higher than their nonketamine counterparts [7, 24, 25]. Nonetheless, 42% of ketamine patients in our study had a reported incidence of agitation or delirium as compared to 21% of propofol patients (*p*=0.04). Given the retrospective nature of this study, we were not able to fully elucidate whether this higher incidence of delirium and agitation was strictly due to the use of ketamine or secondary to hyperactive delirium in critical illness. The use of the Confusion Assessment Method for the ICU scale was not feasible in most MV patients due to their sedation levels. Our study is not without limitations. Given its retrospective nature at a single academic medical center, we were limited with our internal data available through our electronic medical record. The timepoints of interest were determined by the closest snapshot possible to the desired interval found in the chart and were not always precise. The higher APACHE-II score and proportion of concurrent dexmedetomidine use in the ketamine group were two other major limitations. Additionally, we experienced a high mortality rate during this surge of the pandemic. This limited the potentiality of evaluating other clinical outcomes such as time to extubation and the true incidence of delirium during and after ketamine infusion. The complex patient acuity coupled with a high “patient-to-nursing” ratio also restricted their ability to promptly assess and titrate vasopressor doses when appropriate. It would also have been interesting to evaluate the impact of ketamine infusion and the development of cholestatic liver injury as recently reported by Wendel-Garcia and colleagues [26]. Data on remdesivir, tocilizumab, and dexamethasone specifically were not captured in the study due to the evolving standard of care and would likely affect outcomes. Data regarding proning, exposure to neuromuscular blockers, and concurrent exposure to benzodiazepines were simply not collected and may also contribute to differences seen between the groups.

## 5. Conclusion

In this study, we described our experience of using ketamine as continuous infusion in MV patients with SARS-CoV-2 in comparison to propofol. Ketamine was not associated with a decrease in vasopressor requirements at 24 h, 48 h, and 72 h postinitiation of sedation as compared to propofol. However, at select timepoints, this agent was associated with a significant increase in MAP as well as a decrease in opioid requirements and CRP levels. The true utility of using ketamine as a potential anti-inflammatory agent in SARS-CoV-2 infection remains highly exploratory and warrants future studies.

## Figures and Tables

**Table 1:** Baseline characteristics.

Characteristic	Ketamine *N* = 31	Propofol *N* = 53	*P* value
Male sex: *n* (%)^*∗*^	23 (74.2)	34 (64.2)	0.34
Age (years): mean ± SD^*∗∗*^	61.16 ± 11.4	60.5 ± 14.4	0.84
BMI (kg/m^2^): mean ± SD^*∗∗*^	31.51 ± 6.9	32.34 ± 7.5	0.62
Comorbidities: *n* (%)^*∗*^			
Hypertension	17 (54.8)	32 (60.4)	0.62
Diabetes	15 (48.4)	28 (52.8)	0.69
Obesity	21 (67.7)	32 (61.5)	0.57
Atrial fibrillation	1 (3.2)	1 (1.9)	0.70
Coronary artery disease	4 (12.9)	5 (9.4)	0.62
Pulmonary disease	2 (6.5)	5 (9.4)	0.63
Psychiatric disease	0 (0)	1 (1.9)	0.44
Concurrent therapy–*n* (%)^*∗*^			
Dexmedetomidine	19 (61.3)	15 (28.3)	0.003
Systemic corticosteroid	30 (96.8)	49 (92.5)	0.42
Vasopressor	21 (67.7)	24 (45.3)	0.046
APACHE II score: mean ± SD^*∗∗*^	24.58 ± 6.7	21.60 ± 4.6	0.017
Baseline MAP (mmHg): mean ± SD^*∗∗*^	89.90 ± 15.3	84.50 ± 15.1	0.12
Baseline CRP (mg/dL): median (IQR)^ǂ^	14.7 (6.49–22)	13 (7.9–19.3)	0.71
Baseline ferritin (mcg/L): median (IQR)^ǂ^	1127.8 (730–1517)	997 (534–1783)	0.89
Sedative dose (mg/min for ketamine and mcg/kg/min for propofol) (mean ± SD)^ǂǂ^			N/A
Baseline	0.77 ± 1.1	14.17 ± 12.5	
24 h	2.10 ± 1.2	31.70 ± 15.1	
48 h	2.37 ± 1.3	32.26 ± 12.4	
72 h	2.37 ± 1.2	34.72 ± 14.2	
Desired RASS goal at the baseline: *n* (%)^*∗*^			0.14
−5	18 (58.1)	15 (28.3)	
−4	7 (22.6)	20 (37.7)	
−3	1 (3.2)	5 (9.4)	
−2	4 (12.9)	11 (20.8)	
−1	1 (3.2)	1 (1.9)	
0	0 (0.0)	1 (1.9)	
Baseline vasopressor dose in NE (mcg/min): median (IQR)^ǂ^	1 (0–9)	0 (0–3)	0.12
Duration of sedative (hour): mean ± SD^*∗∗*^	154.41 ± 96.4	179.71 ± 100.7	0.26
Baseline opioid infusion rate in ME (mg/hr): median (IQR)^ǂ^	0 (0–25)	2.5 (2.5–10)	0.451

SD: standard deviation, BMI: body mass index, APACHE II: Acute Physiology and Chronic Health Evaluation II, MAP: mean arterial pressure, CRP: C-reactive protein, IQR: interquartile range, RASS: Richmond Agitation Sedation Scale, NE: norepinephrine equivalent, ME: morphine equivalent. ^*∗*^Chi-squared test. ^*∗∗*^Two-sided Student's *t*-test for parametric data. ^ǂ^Wilcoxon rank-sum test for nonparametric data. ^ǂǂ^Noncomparable data given different dosing regimens of propofol and ketamine.

**Table 2:** Primary outcome.

	Ketamine *N* = 31	Propofol *N* = 53	*P* value
Vasopressor requirement (mcg/min): median (IQR)^ǂ^			
24 h	3 (0–16)	6 (0–16.5)	0.35
48 h	4 (0–33)	4 (0–14)	0.78
72 h	6 (0–11)	4 (0–24)	0.73

IQR: interquartile range. ^ǂ^Wilcoxon rank-sum test for nonparametric data.

**Table 3:** Secondary outcomes.

Secondary outcomes	Ketamine *N* = 31	Propofol *N* = 53	*P* value
MAP (mmHg): mean ± SD^*∗∗*^			
24 h	79.87 ± 11.60	72.58 ± 9.67	0.001
48 h	78.12 ± 13.44	72.90 ± 11.18	0.03
72 h	72.64 ± 11.80	74.67 ± 8.21	0.82
96 h	81.72 ± 10.95	71.45 ± 9.60	<0.0001
Average continuous opioid infusion in ME (mg/hr): median (IQR)^ǂ^	3 (0–16)	12.5 (7–16.5)	0.02
Total intermittent opioid amount in ME (mg): median (IQR)^ǂ^	13.84 (3.8–26.9)	7.5 (2.5–10)	0.25
Agitation–*n* (%)^*∗*^	13 (41.94)	11 (20.75)	0.04
Richmond agitation sedation scale compliance: *n* (%)^*∗*^			
24 h	23 (74.2)	36 (67.9)	0.54
48 h	26 (83.9)	42 (79.3)	0.60
72 h	27 (87.1)	42 (79.3)	0.37
CRP (mg/dL): median (IQR)^ǂ^			
24 h	7.53 (1.48–19.7)	15.9 (10.04–21.8)	0.03
48 h	5.23 (1.19–17.65)	14.1 (8.11–26.2)	0.0083
72 h	6.4 (1.74–13.25)	12.1 (8.5–21.4)	0.0085
Ferritin (mcg/L): median (IQR)^ǂ^			
24 h	868 (544–1291)	1115 (702–2132)	0.13
48 h	929 (438–1218)	1091 (526–1848)	0.30
72 h	977 (421–1278)	1143 (467–2013)	0.14
ICU LOS (day): median (IQR)^ǂ^	18 (12–30)	9 (6–14)	0.0002
Mortality: *n* (%)^*∗*^	28 (90.3)	52 (98.1)	0.106

MAP: mean arterial pressure, SD: standard deviation, ME: morphine equivalent, IQR: interquartile range, CRP: C-reactive protein, ICU: intensive care unit, LOS: length of stay. ^*∗*^Chi-squared test. ^*∗∗*^Two-sided Student's *t*-test for parametric data. ^ǂ^Wilcoxon rank-sum test for nonparametric data.

**Table 4:** Select multivariate linear regression analyses^*∗*^.

Outcome of interest	Regression coefficient [95% CI]	*P* value
*MAP (mmHg) at 24 h*		
Ketamine group	8.413 [2.96–13.86]	0.003
Agitation	2.305 [−2.99–7.61]	0.39
Baseline dexmedetomidine use	−2.309 [−7.51–2.89]	0.38
Baseline vasopressor dose (squared)^*∗∗*^	−0.076 [−1.80–1.65]	0.93
APACHE II score (log)^*∗∗*^	−2.180 [−12.78–8.42]	0.68

*CRP (mg/dL) at 24 h*		
Ketamine group	−5.476 [−11.86–0.91]	0.09
Concurrent use of dexmedetomidine	−0.0134 [−6.52–6.25]	0.96

*CRP (mg/dL) at 48 h*		
Ketamine group	−9.533 [−16.02–(−3.04)]	0.005
Concurrent use of dexmedetomidine	4.740 [−1.69–11.18]	0.14

*CRP (mg/dL) at 72 h*		
Ketamine group	2.62 [−10.82–(−0.033)]	0.038
Concurrent use of dexmedetomidine	2.60 [−5.97–4.44]	0.77

CI: confidence interval, MAP: mean arterial pressure, APACHE-II: Acute Physiology and Chronic Health Evaluation II, CRP: C-reactive protein. ^*∗*^Complete results of all multivariate linear regression analyses can be found in the supplemental materials. ^*∗∗*^To allow for model fitting when analyzing multivariate regression analyses.

## Data Availability

The data used to support the findings of this study are included within the article.
