# Supplementary material for: Continuous Infusion of Ketamine in Mechanically Ventilated Patients with SARS-CoV-2
Source: Crit Care Res Pract. 2024 May 10;2024:7765932. doi: 10.1155/2024/7765932 (PMC11101250; doi:10.1155/2024/7765932)
Supplement: Supplementary Materials — Complete sets of these analyses can be found in the supplemental materials. [file 7765932.f1.docx]

| **Outcome of interest** | **Regression Coefficient [95% CI]** | **P-value** |
| --- | --- | --- |
| **Vasopressor Requirement at 24h**  Ketamine group  Agitation  Baseline dexmedetomidine use  Baseline vasopressor dose (squared)*  APACHE II score (log)*  **Vasopressor Requirement at 48h**  Ketamine group  Agitation  Baseline dexmedetomidine use  Baseline vasopressor dose (squared)*  APACHE II score (log)*  **Vasopressor Requirement at 72h**  Ketamine group  Agitation  Baseline dexmedetomidine use  Baseline vasopressor dose (squared)*  APACHE II score (log)* | 0.171 [-9.11 – 9.45]  -8.928 [-17.97 – 0.11]  -0.767 [-9.63 – 8.09]  5.164 [2.22 – 8.10]  2.454 [-15.62 – 20.53]  1.822 [-8.41 – 12.06]  -7.631 [-17.59 – 2.33]  8.535 [-1.24 – 18.31]  3.151 [-0.09 – 6.39]  19.426 [-0.49 – 39.34]  -1.788 [-13.74 – 10.16]  -7.110 [-18.75 – 4.52]  10.562 [-0.85 – 21.98]  3.689 [-0.09 – 7.47]  -1.896 [-25.16 – 21.37] | 0.04  0.05  0.86  0.001  0.78  0.72  0.13  0.09  0.06  0.06  0.77  0.23  0.07  0.06  0.87 |
| **MAP (mmHg) at 24h**  Ketamine group  Agitation  Baseline dexmedetomidine use  Baseline vasopressor dose (squared)*  APACHE II score (log)*  **MAP (mmHg) at 48h**  Ketamine group  Agitation  Baseline dexmedetomidine use  Baseline vasopressor dose (squared)*  APACHE II score (log)*  **MAP (mmHg) at 72h**  Ketamine group  Agitation  Baseline dexmedetomidine use  Baseline vasopressor dose (squared)*  APACHE II score (log)* | 8.413 [2.96 – 13.86]  2.305 [-2.99 – 7.61]  -2.309 [-7.51 – 2.89]  -0.076 [-1.80 – 1.65]  -2.180 [-12.78 – 8.42]  3.999 [-2.03 – 10.03]  3.354 [-2.61 – 9.31]  -2.002 [-7.64 – 3.63]  -1.433 [-2.49 – (-0.37)]  3.183 [-8.28 – 14.65]  -3.479 [-8.25 – 1.29]  2.069 [-2.63 – 6.77]  3.344 [-1.15 – 7.84]  -1.202 [-2.01 – (-0.39)]  2.188 [-7.17 – 11.55 | 0.003  0.39  0.38  0.93  0.68  0.19  0.27  0.48  0.009  0.58  0.15  0.38  0.14  0.004  0.64 |
| **CRP (mg/dL) at 24h**  Ketamine group  Concurrent use of dexmedetomidine  **CRP (mg/dL) at 48h**  Ketamine group  Concurrent use of dexmedetomidine  **CRP (mg/dL) at 72h**  Ketamine group  Concurrent use of dexmedetomidine | -5.476 [-11.86 – 0.91]  -0.0134 [-6.52 – 6.25]  -9.533 [-16.02 – (-3.04)]  4.740 [-1.69 – 11.18]  2.62 [-10.82 – (-0.033)]  2.60 [-5.97 – 4.44] | 0.09  0.96  0.005  0.14  0.038  0.77 |

**To allow for model fitting when analyzing multivariate regression analyses
